# A rapid, sensitive, and field-deployable RAA-EsCas13d platform for detection of porcine adenovirus type 3

**DOI:** 10.1128/spectrum.01291-26

**Published:** 2026-08-14

**Authors:** You-you Li, Hai Zhang, Li-na Shao, Bao-ling Liu, Yuan-meng Wang, Jia-qi Duan, Si-Yuan Lai, Zhi-wen Xu, Ling Zhu

**Affiliations:** 1 Sichuan Key Laboratory of Animal Epidemic Disease and Human Health, College of Veterinary Medicine, Sichuan Agricultural University 506176, Chengdu, China; 2 Sichuan Water Conservancy Vocational College 654679 , Chengdu, China; Children's National Hospital, George Washington University, Washington, DC, USA

**Keywords:** RAA, CRISPR/EsCas13d, single-step detection, extraction-free

## Abstract

Rapid, sensitive, and RADIANT-deployable nucleic acid diagnostics are essential for the prevention and control of porcine adenovirus type 3 (PAdV-3). In this study, we developed RADIANT (RAA-Cas13d DIagnostic plATform for extractioN-free Testing), an extraction-free platform capable of delivering accurate and portable detection in resource-limited settings. By systematically optimizing the reaction buffer composition, recombinase-aided amplification (RAA), T7 transcription, and CRISPR/EsCas13d-mediated cleavage were integrated into a one-pot reaction, reducing the total time-to-result to within 30 minutes. A simplified nucleic acid release step eliminated the need for laboratory-based extraction or complex heating, enhancing the accessibility of the assay. Incorporation of lyophilized reagents minimized cold-chain requirements and simplified assay preparation, enabling cost-effective storage and transport. The platform demonstrated high sensitivity in detecting PAdV-3 and offered two straightforward readout options, fluorescence under 470 nm blue/UV light and lateral flow assay (LFA), facilitating flexible interpretation under diverse RADIANT conditions. Validation with 56 clinical samples showed 100% concordance with quantitative PCR, confirming RADIANT as a rapid, user-friendly, and reliable on-site diagnostic tool for PAdV-3 detection.

**IMPORTANCE:**

Porcine adenovirus type 3 (PAdV-3) is an important swine pathogen for which rapid, practical, and field-compatible diagnostic tools are still lacking. Here, we developed RADIANT, a CRISPR/EsCas13d-based platform that expands PAdV-3 detection beyond conventional laboratory workflows and supports accessible molecular testing in resource-limited settings. Its simple operation and adaptable visual readouts enhance its suitability for on-site use without compromising reliability. This study provides a valuable diagnostic approach for PAdV-3 surveillance and disease control and supports the broader application of portable CRISPR-based technologies in veterinary diagnostics.

## INTRODUCTION

In recent years, outbreaks of various infectious diseases in humans and animals, such as SARS-CoV-2, Zika virus (ZIKV), and monkeypox virus (MPXV), have imposed heavy burdens on global health and the economy (1–3). Rapid and highly specific diagnosis is particularly important for the prevention and control of these diseases. Quantitative polymerase chain reaction (qPCR) is currently considered the gold standard for viral detection, owing to its high specificity and sensitivity (4–6). However, it is limited by centralized sampling, prolonged turnaround time, and reliance on laboratory infrastructure. Point-of-care testing (POCT) plays a critical role in addressing these challenges (7, 8). Isothermal nucleic acid amplification technologies, including nucleic acid sequence-based amplification (NASBA), loop-mediated isothermal amplification (LAMP), transcription-mediated amplification (TMA), strand displacement amplification (SDA), helicase-dependent amplification (HDA), recombinase-aided amplification (RAA), and recombinase polymerase amplification (RPA), enable rapid nucleic acid amplification at a constant temperature, thereby reducing dependence on thermal cyclers and shortening assay turnaround time (9–15). These methods are generally rapid, efficient, and compatible with simple heating devices and diverse visual readouts, making them attractive for point-of-care and field-deployable diagnostics (9–15). Nevertheless, isothermal amplification also has important limitations, including complex primer design in some systems, such as LAMP, potential specificity loss in low-temperature amplification methods, such as NASBA, primer-dependent nonspecific amplification, and increased contamination risk caused by highly efficient amplification (9–15). These limitations may compromise assay specificity and reliability, particularly in visual readout formats. Therefore, coupling isothermal amplification with highly specific CRISPR/Cas-mediated target recognition provides an effective strategy to improve diagnostic specificity while maintaining rapid and field-compatible detection.

Recently, CRISPR/Cas-based diagnostics (CRISPR-Dx) have emerged as promising tools for POCT because of their high specificity and simple reaction conditions (16). However, direct CRISPR-based pathogen detection is generally much less sensitive than qPCR (17), and is therefore often combined with isothermal amplification (18, 19). Classical platforms, such as SHERLOCK and DETECTR, rely on two-step workflows, which increase assay time, operational complexity, and contamination risk (20, 21). To address these limitations, one-pot strategies integrating amplification and CRISPR cleavage have been developed (13, 22). Nevertheless, Cas12-based one-pot systems often suffer from reduced sensitivity due to competition with RPA for reaction templates (23). In addition, the PAM requirement of Cas12 and the PFS requirement of Cas13a further restrict guide RNA design for highly variable viral targets (19, 24–27).

EsCas13d is a compact RNA-targeting CRISPR effector with advantages for diagnostics, including easier purification and greater crRNA design flexibility due to the absence of PAM or PFS constraints (28, 29). However, current Cas13d-based assays still face practical limitations, including complex workflows, insufficient one-pot integration, limited reagent stabilization, and cumbersome sample preparation (30, 31).

Porcine adenovirus type 3 (PAdV-3) is a widely distributed pathogen in swine populations and has been associated with respiratory and gastrointestinal disorders, particularly in young pigs (32). Although infection is often subclinical, PAdV-3 may act as a co-infecting agent that aggravates disease severity and contributes to economic losses in the swine industry. Its frequent occurrence in asymptomatic or mixed infections complicates clinical diagnosis, highlighting the need for rapid, sensitive, and field-deployable detection methods for timely surveillance and disease control.

To address these limitations, we developed RADIANT (RAA-Cas13d DIagnostic plATform for extractioN-free Testing), a one-pot detection system for rapid, sensitive, and extraction-free detection of PAdV-3. RADIANT integrates RAA amplification with EsCas13d-mediated detection in a single tube, thereby reducing operational complexity and shortening detection time. The platform leverages room-temperature lysis buffers to accelerate the reaction without requiring thermal control. Incorporation of lyophilized reagents further enhances portability and storage stability. While maintaining high accuracy, these improvements reduce operational costs and enable application in resource-limited settings. We demonstrate that RADIANT can directly detect PAdV-3 from samples at ambient temperature and supports multiple readout formats, including fluorescence, lateral flow strips, and tube-based visual detection.

## MATERIALS AND METHODS

### Sample collection and viral lysis

To systematically evaluate the clinical performance of the single-step RADIANT platform, a total of 56 clinical samples suspected of PAdV-3 infection were collected from multiple cities in Sichuan Province, China. To minimize potential selection bias, these specimens were collected as prospectively obtained clinical samples with unknown PAdV-3 infection status at the time of sample collection and RADIANT testing, rather than as pre-characterized positive or negative specimens. Sample inclusion was based on clinical suspicion of PAdV-3 infection, including respiratory or gastrointestinal signs, such as diarrhea, respiratory distress, or poor growth performance, together with sample availability and sufficient sample volume for parallel RADIANT and qPCR analysis. No samples were selected or excluded according to prior qPCR results. Samples with insufficient volume, incomplete sample information, or severe contamination were excluded from the analysis. The sampling locations included Chengdu, Ziyang, Guang’an, Ya’an, Meishan, Dazhou, Mianyang, and Nanchong. Sample types included rectal swabs, fecal swabs, and oral swabs. Detailed information on sample origin, collection time, and sample type is provided in Table S1. In addition, five clinical samples positive for porcine reproductive and respiratory syndrome virus (PRRSV), porcine epidemic diarrhea virus (PEDV), Getah virus (GETV), Japanese encephalitis virus (JEV), and pseudorabies virus (PRV), respectively, were included to evaluate the analytical specificity of the platform.

A commercial nucleic acid extraction kit (FastPure Viral DNA/RNA Mini Kit, Vazyme, Nanjing, China) in combination with qPCR was used as the reference standard for evaluating the performance of the RAA-EsCas13d assay. The qPCR assay was performed according to the previously reported real-time PCR method established by Xie et al. (33).

For extraction-free sample processing, a commercial nucleic acid release reagent (Tiosbio, Beijing, China) was used to rapidly release viral nucleic acids at room temperature within 5 min. Briefly, rectal swabs, fecal swabs, and oral swabs were each mixed with the release reagent at a volume ratio of 4:1. The resulting lysates were then directly transferred into the RADIANT detection system for subsequent analysis.

### Expression and purification of EsCas13d

The pET28a-MH6-EsCas13d expression plasmid was obtained from Addgene (Addgene, Plasmid #108303, MA, USA). EsCas13d protein was expressed and purified, as previously described (29). Protein expression was verified by SDS–PAGE. The purified protein was stored at a final concentration of 2 mg/mL in storage buffer (50 mM Tris-HCl, 1 M NaCl, 10% glycerol, 2 mM DTT).

### Verification of EsCas13d cleavage activity

The 20 μL EsCas13d cleavage reaction mixture consisted of Buffer I (20 mM HEPES, pH 7.1, 50 mM KCl, 5 mM MgCl_2_, and 5% glycerol), 50 nM EsCas13d protein, 100 nM crRNA, 1 × 10^6^ copies/μL ssRNA target, 2 U/μL RNase inhibitor, and 1 μM fluorescent RNA reporter (FQ-ssRNA). The reactions were incubated at 37°C for 30 min in a QuantStudio 1 Plus Real-Time PCR System (Thermo Fisher Scientific, MA, USA), and fluorescence signals were recorded every 30 s. Unless otherwise specified, fluorescence was measured once every 30 s.

To evaluate the nuclease activity of EsCas13d, five reaction conditions were tested: (i) a complete reaction containing all components, (ii) a reaction lacking EsCas13d protein, (iii) a reaction lacking crRNA, (iv) a reaction lacking the target RNA, and (v) a reaction lacking Mg^2+^.

### Screening of RAA primers and crRNA

Using the PAdV-3 genome as the target, nine pairs of RAA primers and three guide DNA (gDNA) sequences were designed based on the highly conserved region of the E3 gene. Two ssRNA reporters were also synthesized, including a fluorescent reporter (Probe-1) and a reporter for lateral flow assay (LFA) detection (Probe-2) (Table S2). All oligonucleotides were synthesized by Sangon Biotech (Shanghai, China) and stored at −20°C until use.

For crRNA preparation, each gDNA oligonucleotide was mixed with an equimolar amount of the T7 promoter oligonucleotide, denatured at 95°C for 5 min, and then gradually cooled to 20°C at a rate of 0.1°C/s to generate double-stranded DNA templates. *In vitro* transcription was carried out at 37°C for 4 h using a HiScribe T7 High Yield RNA Synthesis Kit. The resulting crRNAs were purified using a column-based RNA rapid purification kit and stored at −80°C until use. To facilitate subsequent *in vitro* transcription of the RAA amplicons, the T7 promoter sequence (5′-TAATACGACTCACTATAGGG-3′) was appended to the 5′ end of each forward primer. The three crRNAs were screened using the EsCas13d cleavage assay described in the section “Verification of EsCas13d cleavage activity.” For RAA primer screening, basic lyophilized RAA pellets were used according to the manufacturer’s instructions, and amplification reactions were carried out at 37°C for 20 min. The resulting amplicons were first analyzed by agarose gel electrophoresis. In parallel, the amplification products were subjected to *in vitro* transcription, and the resulting transcripts were introduced into the EsCas13d detection system for fluorescence measurement to further evaluate primer performance.

Sequence information for the synthetic targets, RAA primers, T7 promoter, and gDNAs is provided in Table S2.

### Two-step RAA-CRISPR/EsCas13d assay

The two-step detection workflow consisted of an initial RAA amplification step followed by an *in vitro* transcription-coupled EsCas13d cleavage reaction. In the first step, the RAA reaction mixture contained 400 nM forward and reverse primers, 14 mM magnesium acetate, RNase inhibitor, and template nucleic acid accounting for 10% of the total reaction volume. The reaction was incubated at 37°C for 20 min.

The second-step reaction mixture consisted of Buffer I (20 mM HEPES, pH 7.1, 50 mM KCl, 5 mM MgCl₂, and 5% glycerol), 65 nM EsCas13d, 100 nM crRNA, 2 mM rNTPs, 1 U/μL T7 RNA polymerase, 0.04 U/μL RNase H, and 1 μM fluorescent reporter (FQ-ssRNA). The product from the first-step RAA reaction was then added to the second-step reaction at a volume ratio of 5%, followed by incubation at 37°C for 30 min. Fluorescence signals were recorded using a QuantStudio 1 Plus Real-Time PCR System.

### Establishment of single-step RADIANT

At present, only limited studies have reported one-pot platforms integrating isothermal amplification with EsCas13d-based detection, and no RAA-EsCas13d single-step system has been described to date. To explore a reaction buffer suitable for one-pot RAA-EsCas13d detection, we first adapted the single-step reaction buffer previously developed for the LwaCas13a-based SHINE platform (designated Buffer II) and evaluated its compatibility with the RAA-EsCas13d system (16).

The Buffer II-based reaction mixture consisted of 20 mM HEPES (pH 8.0), 50 mM KCl, 5% PEG-8000, 65 nM EsCas13d, 100 nM crRNA, 1 μM FQ reporter, 2 U/μL RNase inhibitor, 0.02 U/μL RNase H, 1.2 U/μL T7 RNA polymerase, 2 mM rNTPs, 0.4 μM forward and reverse primers, and 14 mM magnesium acetate. Template DNA was mixed with the reaction system at a ratio of 1:19, and the reaction was incubated at 37°C for 30 min with fluorescence monitoring.

To optimize the one-pot RAA-EsCas13d system, key reaction parameters, including pH, KCl concentration, Mg²^+^ concentration, and primer concentration, were sequentially adjusted. Based on these optimization results, the final reaction formulation (Buffer III) was established and used for subsequent assays. The analytical specificity of the platform was evaluated using nucleic acids from porcine viruses other than PAdV-3 as negative controls. The limit of detection was determined using serially diluted standard templates and was compared with those of the two-step assay and the unoptimized one-pot system.

### Visualization of RADIANT assay

For in-tube fluorescence detection, the reaction master mix was prepared as described above, and samples were added at a volume ratio of 1:19. The reaction mixture was incubated at 37°C for 30 min. After incubation, the reaction tubes were immediately visualized under a UV transilluminator.

For lateral flow assay (LFA) readout, the reaction mixture was identical to that used for fluorescence detection, except that the quenched ssRNA reporter was replaced with a FAM-biotin poly-U reporter. Following isothermal incubation, a commercial lateral flow strip (Tulugang, Suzhou, China) was directly inserted into the reaction tube with the absorbent pad fully immersed. For LFA interpretation, the appearance of both the control line and test line within the observation window was classified as a positive result, whereas the appearance of only the control line was classified as a negative result. Tests without a visible control line were considered invalid and were repeated. Because weak test lines may occur in samples with low target concentrations, all LFA strips were interpreted independently by three observers who were blinded to the qPCR results. A sample was classified as LFA-positive when the test line was judged to be visible by at least two of the three observers. Discrepant readings, if any, were resolved by repeat testing and comparison with the corresponding fluorescence readout.

In this format, the FAM-labeled end of the intact reporter binds to the gold nanoparticle conjugate coated with anti-FAM antibodies on the strip, while the biotin-labeled end is captured at the streptavidin-coated control line. Therefore, when Cas13d-mediated cleavage does not occur, only the control line is visible. In contrast, cleavage of the reporter by activated Cas13d generates FAM-labeled fragments that accumulate at both the test line and the control line, resulting in two visible bands. Unless otherwise specified, both visualization formats were used for result presentation.

### Lyophilized RADIANT assay

Three lyophilization buffers were developed with reference to the SHINE v.2 freeze-drying system: Buffer A (20 mM HEPES, pH 7.0, 50 mM KCl, and 5% PEG-8000), LYO (20 mM HEPES, 5% sucrose, and 150 mM mannitol), and LYO + DTT (LYO supplemented with 10 mM DTT). For lyophilization, TwistAmp reaction pellets and the corresponding reaction master mix were reconstituted using the buffers described above. The mixtures were rapidly frozen in liquid nitrogen and then lyophilized at −40°C for 16–20 h. The resulting freeze-dried pellets were sealed and stored until use. Before detection, the reaction system was reconstituted by adding magnesium acetate and the remaining required components, and samples were added at a ratio of 1:19.

For stability evaluation, lyophilized RADIANT pellets were prepared using the optimized LYOO buffer, as described above. The pellets were stored at room temperature, 4°C, and −20°C for 7, 14, 21, and 28 days prior to performance testing.

### Clinical sample analysis

To systematically evaluate the clinical applicability of the single-step RADIANT platform, 56 clinical samples suspected of PAdV-3 infection were collected and analyzed in this study. All 56 clinical specimens were tested in parallel using RADIANT and qPCR. The qPCR results were used only as the reference standard for performance comparison and were not used for sample preselection. A commercial nucleic acid extraction kit in combination with qPCR was used as the reference standard for comparison to assess the diagnostic performance of RADIANT.

### Statistical analysis

**Fig 1:**
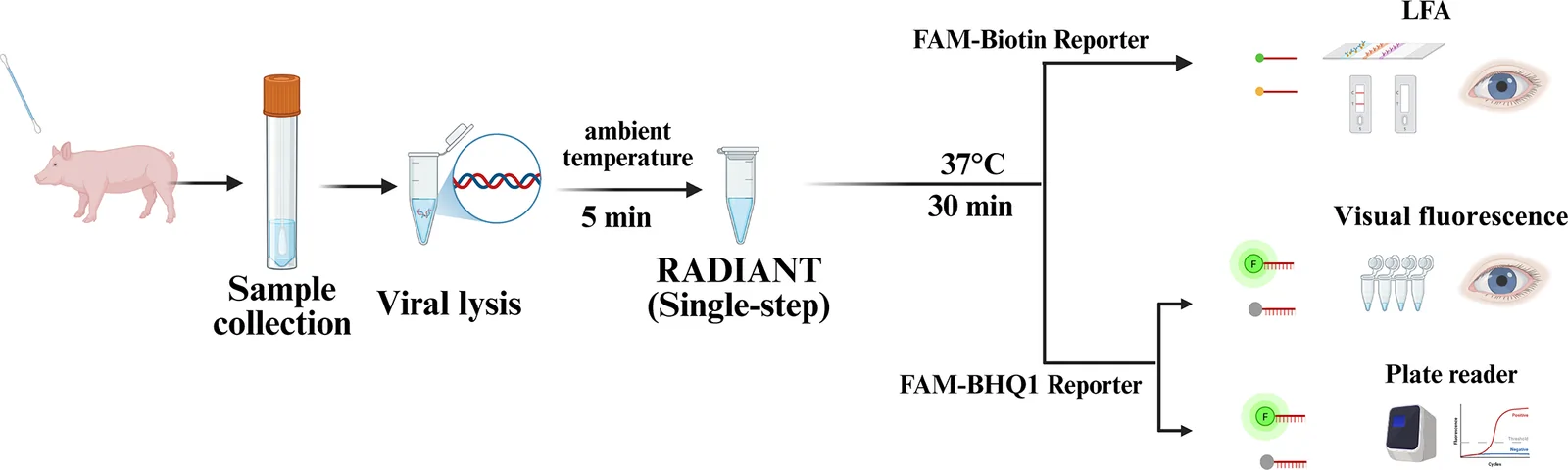
Design principle of the RADIANT platform.

Statistical analyses were conducted using GraphPad Prism 9.0 (GraphPad Software). Experimental data derived from three independent biological replicates are expressed as mean ± standard deviation (SD) for technical reproducibility, with results visualized as mean ± standard error of the mean (SEM) to reflect inter-experimental variability. The schematics shown in Fig. 1 and 5A were created with BioRender.com. All figures were assembled and finalized using Adobe Illustrator 2020.

## RESULTS

### The principle of RADIANT detection

RADIANT was established as a one-pot nucleic acid detection platform based on RAA and EsCas13d, as illustrated in Fig. 1. In this single-tube system, target nucleic acids undergo amplification, transcription, and CRISPR-mediated cleavage in a coordinated reaction. Guided by crRNA, EsCas13d specifically recognizes the target RNA and subsequently cleaves the fluorescently labeled ssRNA reporter, thereby producing a sensitive and specific signal. This integrated assay eliminates the need for conventional nucleic acid extraction and complex instrumentation, enabling rapid detection under isothermal conditions and making it well suited for field deployment. The platform supports three signal readout formats, including real-time fluorescence monitoring with a qPCR instrument, direct visual fluorescence observation in the reaction tube, and LFA-based detection. For in-tube visual fluorescence readout, a FAM-BHQ dual-labeled reporter is used to generate the signal, whereas LFA readout is achieved using a FAM-biotin dual-labeled reporter in combination with a streptavidin-biotin recognition system, allowing instrument-free visual interpretation.

### EsCas13d preparation and screening of core detection components

**Fig 2:**
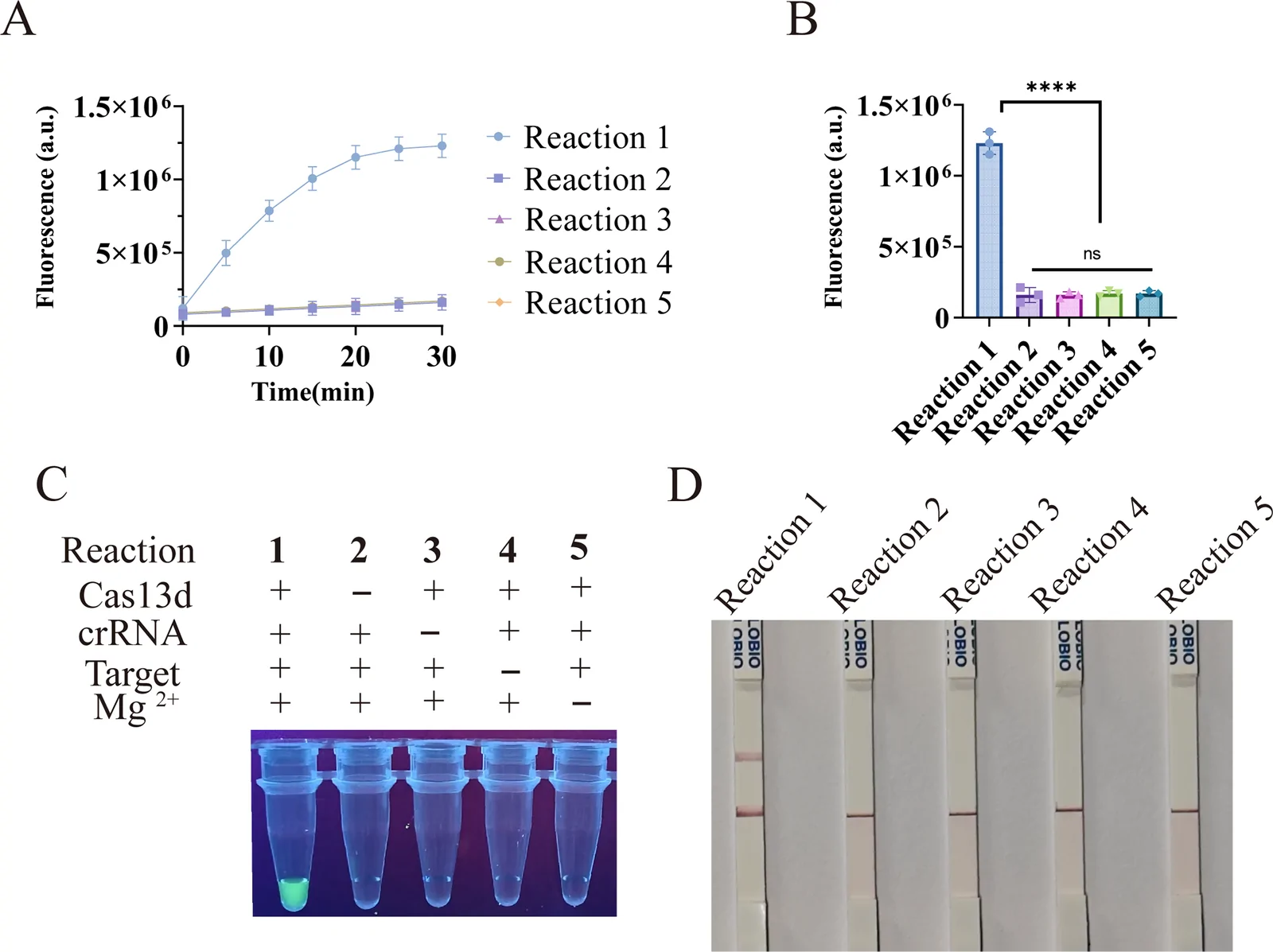
Validation of EsCas13d activity. Five reaction systems were designed to determine the essential components required for EsCas13d activity. “+” indicates the presence of the corresponding component, whereas “−” indicates its absence. Specifically, Reaction 1 contained Cas13d, crRNA, target template, and Mg²^+^; Reaction 2 lacked Cas13d; Reaction 3 lacked Mg²^+^; Reaction 4 lacked crRNA; and Reaction 5 lacked the target template. (**A**) EsCas13d activity was assessed by real-time fluorescence monitoring. (**B**) Endpoint fluorescence intensity further confirmed significant collateral cleavage activity (*n* = 3 independent replicates; error bars indicate SD; ****P < 0.0001; ns, not significant). Visual detection was also performed under (**C**) UV illumination and (**D**) by lateral flow assay (LFA) to further verify EsCas13d-mediated collateral cleavage activity.

**Fig 3:**
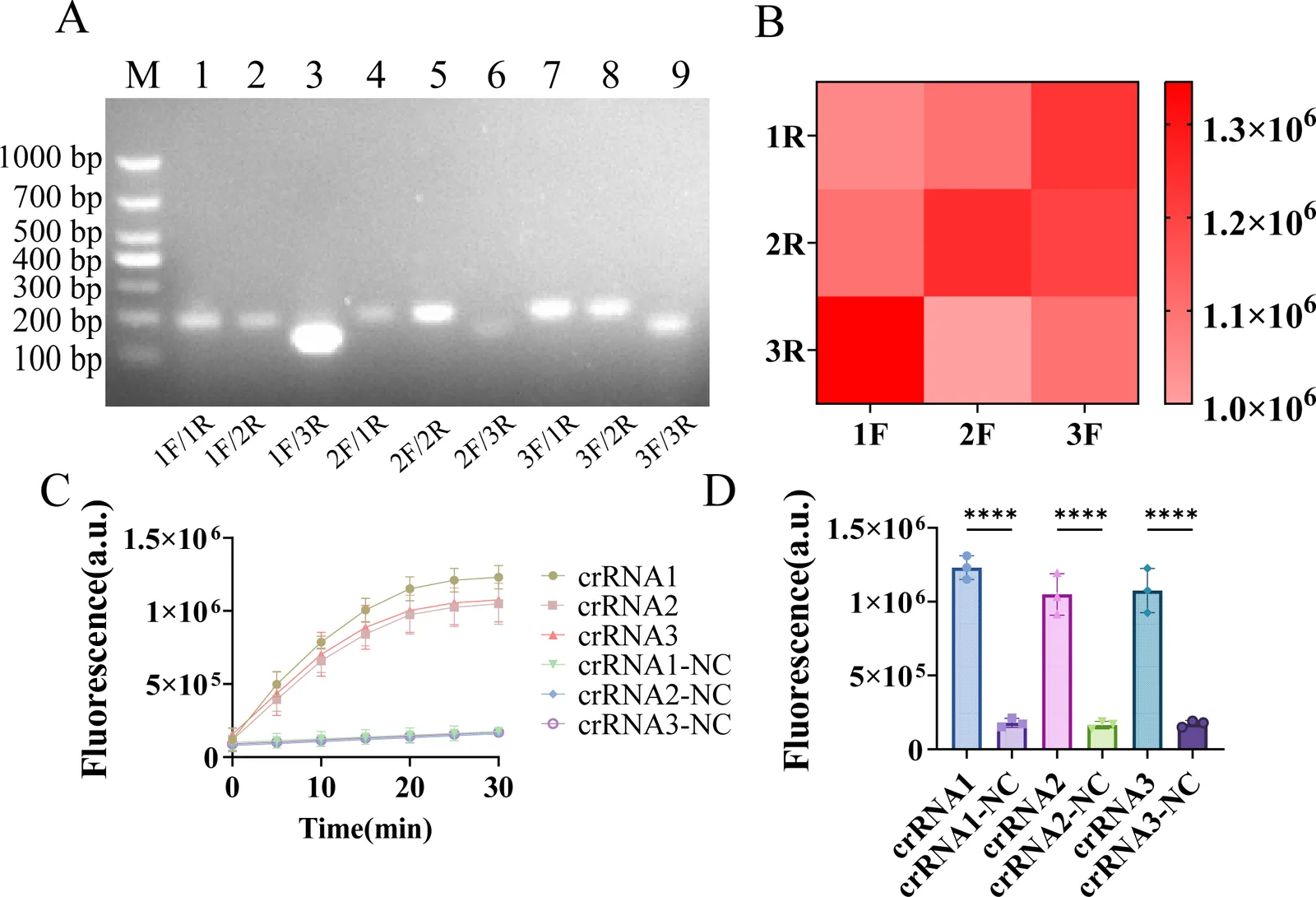
Screening of RAA primers and crRNAs targeting the PAdV-3 E3 gene. (**A**) Agarose gel electrophoresis analysis of amplification products generated by nine pairs of RAA primers targeting the PAdV-3 E3 gene. (**B**) Comparison of fluorescence intensities obtained with the nine primer pairs. (**C**) Real-time fluorescence curves of three crRNAs targeting PAdV-3 E3. (**D**) Comparison of endpoint fluorescence intensities of the three corresponding crRNAs targeting PAdV-3 E3. NC indicates the negative control. ****P < 0.0001; ***P < 0.001; **P < 0.01; *P < 0.05; ns, not significant.

SDS-PAGE analysis confirmed the successful expression and purification of EsCas13d protein (Fig. S1), and subsequent enzymatic assays demonstrated that the purified protein retained robust nuclease activity (Fig. 2). In the complete reaction system containing EsCas13d, crRNA, target RNA, and Mg2+, a clear fluorescence increase was observed, whereas omission of any essential component markedly reduced the fluorescence signal. These results confirmed that the purified EsCas13d protein could specifically recognize the target RNA under crRNA guidance and mediate collateral cleavage of the ssRNA reporter. Based on the high sequence conservation of the E3 gene, nine pairs of RAA primers and three crRNAs were designed for assay development. Preliminary screening by agarose gel electrophoresis, together with the two-step detection assay, identified the 1F/3R primer pair as the optimal amplification set (Fig. 3A and B). Compared with the other primer combinations, the 1F/3R pair generated a clear amplification product and produced the strongest downstream fluorescence signal after transcription-coupled EsCas13d detection, indicating more efficient target amplification and signal generation. The performance of the three crRNAs was then evaluated individually by measuring fluorescence output in the reaction system, and crRNA2 produced the strongest fluorescence signal (Fig. 3C and D). Therefore, the 1F/3R primer pair and crRNA2 were selected as the core detection components for subsequent establishment of the single-step RADIANT assay.

### Development of Single-step RADIANT assay

**Fig 4:**
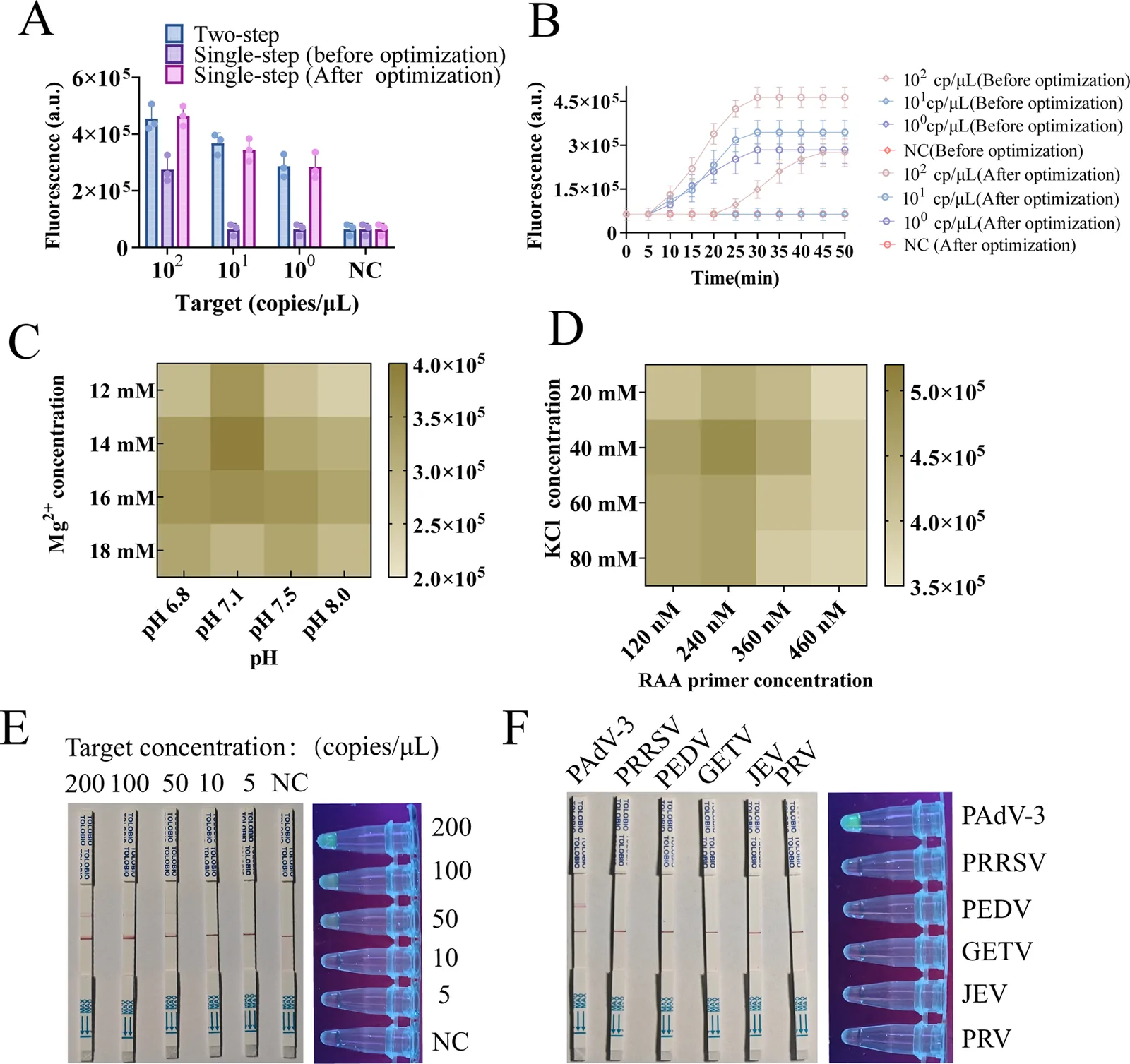
Optimization of the single-step RADIANT platform. (**A**) Comparison of endpoint fluorescence intensities before and after optimization of the one-pot assay. (**B**) Real-time fluorescence curves before and after optimization. (**C**) Heatmap of fluorescence intensities during Mg²^+^ and pH optimization. (**D**) Heatmap of fluorescence intensities during Mg^2+^ and pH optimization. (**E**) Visual detection sensitivity of the optimized RADIANT platform. (**F**) Specificity evaluation of the RADIANT assay.

Our goal was to develop an integrated and simplified detection strategy that would substantially reduce the assay time and operational complexity compared with the conventional two-step RAA-CRISPR/EsCas13d workflow. However, reports on the integration of RAA with EsCas13d remain limited. To establish optimal conditions for the one-pot RAA-EsCas13d assay, we first adapted the single-step reaction buffer previously optimized for the RAA-LwaCas13a system in SHINE v.2 and evaluated the performance of RAA-EsCas13d in this formulation. The results showed that the SHINE v.2 buffer was suboptimal for one-pot RAA-EsCas13d detection, leading to markedly reduced sensitivity and prolonged reaction time (Fig. 4A and B). Specifically, the limit of detection (LoD) was substantially higher than that of the two-step assay, suggesting that the different enzymatic reactions were not fully compatible under the original buffer conditions. Although fluorescence signals could still be detected in the unoptimized one-pot system at relatively high template concentrations, signal accumulation was delayed, and the endpoint fluorescence intensity was reduced, indicating that the initial buffer formulation could not efficiently support RAA amplification, T7 transcription, and EsCas13d-mediated reporter cleavage simultaneously.

To address this limitation, we fixed the input amount of the PAdV-3 genome at a known copy number and sequentially optimized key buffer parameters, including pH, Mg^2+^ concentration, KCl concentration, and primer concentration. Parameter optimization was performed stepwise, with each round based on the previously optimized condition (Fig. 4C and D). Because these components can affect multiple enzymatic steps in the one-pot reaction, each parameter was evaluated according to both fluorescence intensity and signal development kinetics. Ultimately, the optimal reaction conditions were determined to be pH 7.1, 14 mM magnesium acetate, 40 mM KCl, and 240 nM primer concentration (Fig. 4C and D). Under these conditions, the reaction produced stronger fluorescence signals and faster signal accumulation than the unoptimized formulation, suggesting improved biochemical compatibility among the amplification, transcription, and CRISPR detection modules. Based on these results, Buffer III was established as the final optimized formulation, consisting of 20 mM HEPES (pH 7.1), 40 mM KCl, 5% PEG-8000, 65 nM EsCas13d, 100 nM crRNA, 1 μM FQ reporter, 2 U/μL RNase inhibitor, 0.02 U/μL RNase H, 1 U/μL T7 RNA polymerase, 2 mM rNTPs, 0.24 μM each forward and reverse primer, and 14 mM magnesium acetate. Following systematic optimization, the overall reaction time was shortened from 50 min to 30 min (Fig. 4A and B), while the analytical sensitivity was improved to a limit of detection of 10^0^ copy/μL. The total reaction time was defined as the time required for the reaction to reach the plateau phase at the assay’s detection limit. These results demonstrate that buffer optimization was essential for converting the two-step RAA–EsCas13d assay into an efficient single-step RADIANT reaction.

We next evaluated the analytical sensitivity of RADIANT under visual readout conditions. For both in-tube fluorescence visualization and LFA, the signal must reach a sufficient brightness threshold or allow adequate accumulation of colloidal gold particles to enable naked-eye interpretation. Under these visual detection formats, RADIANT was still able to detect PAdV-3 with a sensitivity of up to 50 copies/μL. Clear positive signals were observed in both visual fluorescence and LFA formats at low template concentrations, indicating that the optimized RADIANT assay retained sufficient signal output for instrument-independent interpretation. These findings support the feasibility of applying RADIANT in low-infrastructure or field settings where real-time fluorescence instruments may not be available.

### Lyophilization test results for RADIANT

**Fig 5:**
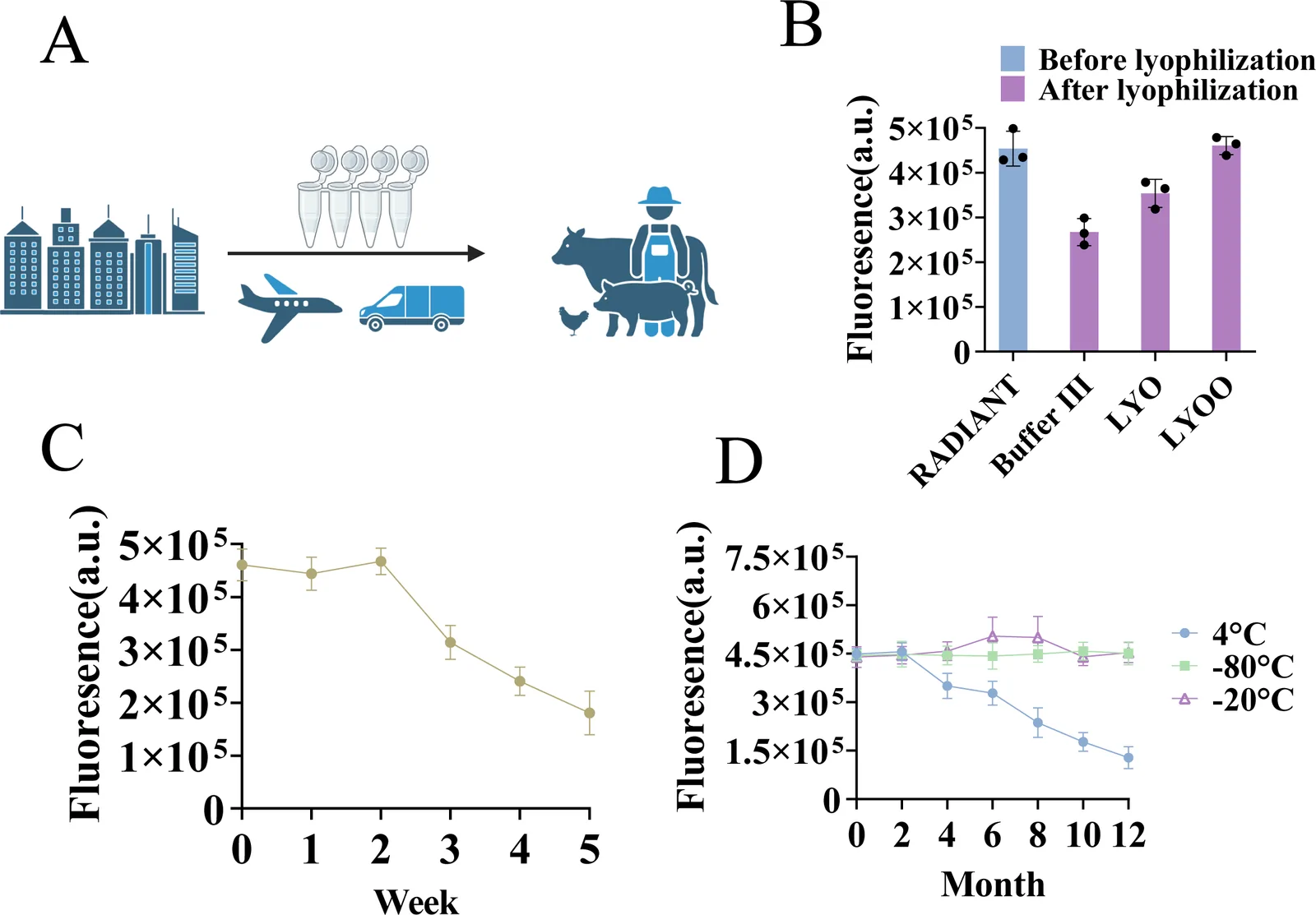
Optimization of lyophilization for the single-step RADIANT platform. (**A**) Schematic illustration of post-lyophilization transportation. (**B**) Comparison of fluorescence intensities obtained with different lyophilization buffers and the assay before lyophilization. (**C**) Storage stability of the lyophilized assay under room-temperature conditions. (**D**) Storage stability of the lyophilized assay at 4°C, −20°C, and −80°C.

Lyophilization reduced the reliance on dry ice during transportation and prolonged reagent shelf life, thereby improving the applicability of RADIANT for POCT (Fig. 5A). It also simplified assay setup by enabling rapid reconstitution of the complete reaction. This format is particularly important for field-deployable diagnostics because it allows the key reaction components to be preassembled and stored in a ready-to-use form, thereby reducing reagent preparation steps before testing. Direct lyophilization of Buffer III resulted in poor detection performance (Fig. 5B). After direct freeze-drying, the fluorescence signal was markedly decreased, indicating that the optimized liquid reaction buffer alone was insufficient to protect the enzymatic components during lyophilization and rehydration. Although the SHINE v.2 lyophilization buffer (LYO) partially improved reagent stability, its performance remained unsatisfactory. To further optimize the formulation, 10 mM DTT was added to LYO to generate LYOO, which showed superior preservation of RADIANT activity after freeze-drying (Fig. 5B). Compared with the other lyophilization formulations, LYOO produced stronger fluorescence recovery after reconstitution, suggesting that the addition of DTT helped maintain the activity of one or more redox-sensitive components in the RAA–T7–EsCas13d reaction system. Using this optimized formulation, the lyophilized reagents remained stable for 2 weeks at 25°C, 2 months at 4°C, and at least 12 months at −20°C and −80°C (Fig. 5C and D). During the storage evaluation, the lyophilized RADIANT pellets retained detectable activity under all tested storage conditions, with better long-term preservation observed at lower temperatures. These results indicate that lyophilization can improve the storage flexibility and transportability of RADIANT, supporting its potential use in decentralized or resource-limited diagnostic settings.

### Clinical sample analysis

**Fig 6:**
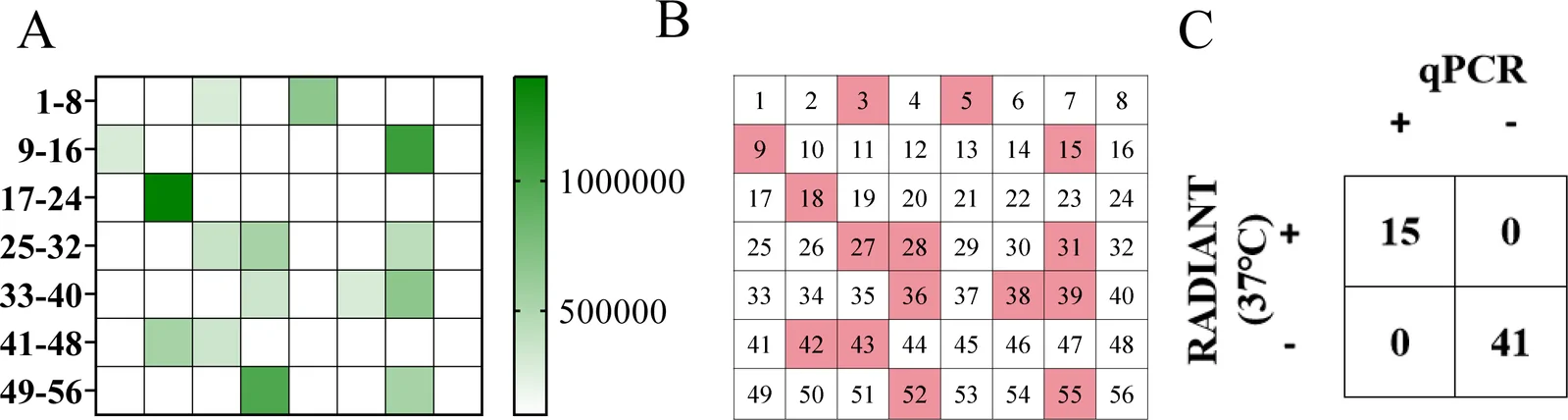
Clinical detection of PAdV-3 in 56 samples by RADIANT and qPCR. (**A**) Detection results of PAdV-3 in 56 clinical samples using the RADIANT assay. (**B**) Detection results of PAdV-3 in the same 56 clinical samples using qPCR. (**C**) Summary comparison of the detection results obtained by RADIANT and qPCR.

To assess the clinical performance of RADIANT, 56 samples suspected of PAdV-3 infection were analyzed. The visual fluorescence and LFA readouts were fully concordant, identifying 15 positive samples, corresponding to a positivity rate of 26.8% (15/56) (Fig. 6A and Fig. S2 and S3). For fluorescence visualization, positive samples produced clearly detectable fluorescence signals under 470 nm blue/UV light, whereas negative samples showed no obvious fluorescence increase. Similarly, in the LFA format, positive samples showed both control and test lines, while negative samples showed only the control line. Further analysis using a commercial nucleic acid extraction kit followed by qPCR yielded identical results (Fig. 6B). All 15 RADIANT-positive samples were confirmed as positive by qPCR, and all 41 RADIANT-negative samples were also negative by qPCR, indicating no false-positive or false-negative results in this sample set. Although several positive samples showed relatively weak test lines on LFA strips, these signals were consistently distinguishable from the negative background by independent observers and were concordant with the corresponding visual fluorescence and qPCR results. This finding suggests that even samples producing relatively low visual LFA signals could still be reliably interpreted when combined with predefined interpretation criteria. The complete agreement between RADIANT and qPCR supports the reliability of the platform for clinical detection of PAdV-3 (Fig. 6C).

## DISCUSSION

CRISPR-Dx has emerged as a promising platform for pathogen detection, with most current systems based on Cas12a or Cas13a (19, 34–36). However, Cas12a is constrained by PAM requirements and reduced efficiency in one-pot reactions due to template competition (35), whereas Cas13a, although avoiding such competition, is limited by its dependence on PFS for target recognition. In addition, both proteins are relatively large, which complicates their expression, purification, and large-scale production. In contrast, Cas13d is smaller and does not require PAM or PFS sequences (37), offering advantages for diagnostic development. Although early studies have demonstrated its potential in pathogen detection, Cas13d-based assays still face challenges, particularly the reliance on two-step workflows and the limited understanding of one-pot reaction chemistry.

In this study, we developed RADIANT, a rapid, sensitive, and field-deployable one-pot nucleic acid detection platform that integrates RAA amplification with EsCas13d-mediated CRISPR detection for the diagnosis of PAdV-3. By coupling target amplification, transcription, and collateral cleavage within a single reaction vessel, RADIANT simplifies the conventional multistep CRISPR-Dx workflow and reduces both hands-on time and the risk of cross-contamination. In the optimized workflow, a brief 5-min nucleic acid release step was followed by a 30-min one-pot reaction, allowing the assay to be completed within approximately 35 min. The platform further supports multiple readout formats, including real-time fluorescence monitoring, in-tube visual fluorescence, and lateral flow assay (LFA), thereby expanding its applicability from laboratory settings to resource-limited and field environments. The agreement among fluorescence visualization, LFA, and qPCR in clinical sample testing further supports the reliability of these readout formats.

A major finding of this study is that direct translation of previously reported one-pot Cas13 reaction conditions to the RAA-EsCas13d system was insufficient to achieve satisfactory performance. Specifically, the SHINE v.2 buffer, which was originally optimized for an RAA-LwaCas13a system, resulted in markedly reduced sensitivity and prolonged reaction time when applied to RAA-EsCas13d. In the initial one-pot formulation, fluorescence accumulation was delayed and the endpoint signal was weaker, indicating that the original buffer could not efficiently support the coordinated operation of RAA amplification, T7 transcription, and EsCas13d-mediated collateral cleavage. This result highlights an important but often underappreciated point in CRISPR-based diagnostics: successful one-pot integration depends not only on the activity of each individual enzyme, but also on the biochemical compatibility among isothermal amplification, transcription, and Cas-mediated cleavage within a shared reaction environment. Because PAdV-3 is a DNA virus, T7-tagged RAA amplicons can be directly transcribed by T7 RNA polymerase to generate target RNA for EsCas13d recognition, making reverse transcription unnecessary in this assay. The poor performance observed in the initial buffer likely reflected suboptimal coordination among these enzymatic steps. Therefore, systematic optimization of the reaction chemistry was essential for establishing a robust RAA-EsCas13d platform.

Through sequential optimization of key reaction parameters, including pH, Mg^2+^ concentration, KCl concentration, and primer concentration, we established Buffer III as the optimal formulation for one-pot RADIANT detection. The optimized conditions were determined to be pH 7.1, 14 mM magnesium acetate, 40 mM KCl, and 240 nM primer concentration. The final optimized reaction conditions significantly improved overall assay performance and reduced the total reaction time from 50 min to 30 min. These findings indicate that relatively small changes in buffer composition can substantially influence the kinetics and sensitivity of one-pot CRISPR assays. In particular, pH and magnesium concentration likely affected multiple stages of the reaction simultaneously, including recombinase-driven amplification, T7 transcription efficiency, and EsCas13d collateral cleavage activity. Similarly, adjustment of ionic strength and primer concentration probably contributed to a better balance between amplification efficiency and downstream CRISPR signal generation. Collectively, these results demonstrate that buffer engineering is a critical step in the development of integrated one-pot diagnostic platforms, especially when adapting Cas13 systems to new amplification chemistries such as RAA.

Another notable feature of RADIANT is its dual visual readout capability. In addition to fluorescence detection using an instrument, the assay could be interpreted directly by in-tube fluorescence or by LFA, both of which are more practical for field use. In the present study, both visual fluorescence and LFA formats enabled direct detection of PAdV-3 without the need for real-time fluorescence instrumentation. As expected, the visual fluorescence and LFA formats exhibited lower sensitivity than fluorescence readout using a plate reader, with both visual methods showing a limit of detection of 50 copies/μL. This difference is reasonable, as signal accumulation sufficient for naked-eye fluorescence observation or formation of a visible test line on LFA strips generally requires a higher output threshold than instrument-based fluorescence detection. Nevertheless, the visual readouts retained adequate analytical performance while offering substantial advantages in portability, simplicity, and instrument independence. Although several clinical LFA-positive samples showed relatively weak test lines, these signals were still distinguishable from the negative background by independent observers and were consistent with the corresponding fluorescence and qPCR results. This trade-off between maximal analytical sensitivity and operational convenience is highly relevant in point-of-care and on-site veterinary testing scenarios.

For practical deployment, reagent stability is as important as analytical performance. In this regard, the lyophilization results further strengthen the translational value of RADIANT. Direct lyophilization of the optimized Buffer III formulation led to poor assay performance, suggesting that the reaction components required additional protection during freeze-drying and storage. Although the SHINE v.2 lyophilization buffer improved stability, its protective effect remained suboptimal. By supplementing this formulation with DTT to generate LYOO, we achieved better preservation of RADIANT activity, indicating that maintenance of a reducing environment is important for protecting one or more key reaction components during lyophilization. Compared with the directly lyophilized Buffer III formulation and the LYO formulation, LYOO showed stronger fluorescence recovery after reconstitution, indicating better preservation of reaction activity. Moreover, the lyophilized reagents exhibited encouraging storage stability over a range of temperatures, including room temperature, refrigeration, and frozen conditions. Specifically, the lyophilized RADIANT reagents remained stable for 2 weeks at 25°C, 2 months at 4°C, and at least 12 months at −20°C and −80°C. These findings are particularly important for decentralized testing, as they reduce reliance on cold-chain transportation, simplify assay setup, and improve the feasibility of distributing complete diagnostic kits to remote settings.

It should be noted that the term “extraction-free” in RADIANT refers to the elimination of conventional laboratory-based nucleic acid extraction and purification procedures, rather than the complete absence of sample processing. In the present workflow, clinical specimens are subjected to a brief nucleic acid release step at room temperature before being added directly to the one-pot reaction. This simplified pretreatment avoids column- or magnetic bead-based extraction, repeated washing or centrifugation, and complex heating equipment, thereby reducing hands-on time and operational requirements. In this study, clinical swab and fecal specimens were mixed with nucleic acid release reagent and processed at room temperature for 5 min, after which the lysates were directly introduced into the RADIANT reaction. Compared with conventional extraction-based workflows, this design improves assay accessibility and is more compatible with field or low-infrastructure diagnostic settings.

Clinical validation further supported the utility of the RADIANT platform. When applied to suspected field samples, both in-tube fluorescence and LFA readouts showed complete agreement with each other and with qPCR-based detection, correctly identifying all positive samples. Among the 56 suspected clinical samples, RADIANT identified 15 PAdV-3-positive samples, corresponding to a positivity rate of 26.8% (15/56). All 15 RADIANT-positive samples were also positive by qPCR, and all 41 RADIANT-negative samples were negative by qPCR. This full concordance suggests that RADIANT provides reliable analytical performance not only under controlled laboratory conditions but also in real clinical specimens. Such consistency is especially meaningful for veterinary diagnostics in field settings, where sample quality may vary and access to sophisticated laboratory infrastructure is limited. Together with its rapid turnaround time, simple workflow, and lyophilized reagent compatibility, this clinical performance highlights the potential of RADIANT as a practical tool for surveillance and early diagnosis of PAdV-3.

### Conclusion

In conclusion, this study established RADIANT, a rapid, sensitive, and field-deployable RAA–EsCas13d platform for the detection of PAdV-3. By integrating RAA amplification, T7 transcription, and EsCas13d-mediated collateral cleavage into a single-tube reaction, RADIANT simplified the detection workflow and enabled rapid nucleic acid detection with visual fluorescence and LFA readouts. The incorporation of a brief extraction-free nucleic acid release step and lyophilized reagents further improved the portability, operational convenience, and storage stability of the assay. Clinical sample evaluation showed complete concordance between RADIANT and qPCR, supporting its potential application for on-site PAdV-3 surveillance and diagnosis in resource-limited settings. Overall, RADIANT provides a practical diagnostic framework that may be further adapted for the rapid detection of other animal pathogens.

## Data Availability

The data are also included in the article and its Supplemental material. Further inquiries can be directed to the corresponding author.

## SUPPLEMENTAL MATERIAL

Tables S1 and S2; Figures S1 to S3 spectrum.01291-26-s0001.docx Table S1: Clinical sample information. Table S2: Nucleotide sequences used in this study. Fig. S1: SDS-PAGE validation of EsCas13d expression and purification. Fig. S2: Visual fluorescence results of clinical samples detected by the RADIANT assay. Fig. S3: LFA detection results of clinical samples using the RADIANT assay.

ASM does not own the copyrights to Supplemental Material that may be linked to, or accessed through, an article. The authors have granted ASM a non-exclusive, world-wide license to publish the Supplemental Material files. Please contact the corresponding author directly for reuse.
