# Supplementary material for: A rapid, sensitive, and field-deployable RAA-EsCas13d platform for detection of porcine adenovirus type 3
Source: Microbiol Spectr. 2026 Aug 14;14(9):e01291-26. doi: 10.1128/spectrum.01291-26 (PMC13532335; doi:10.1128/spectrum.01291-26)
Supplement: Supplementary file 1 [file spectrum.01291-26-s0001.docx]

**Supplementary Table 1. Clinical sample information.**

“+” indicates a positive result, “−” indicates a negative result, and “UN” denotes undetected.

| Sample ID | Sample Type | PAdV-3 | Viral Load(cp/μL) | Sample ID | Sample Type | PAdV-3 | Viral Load(cp/μL) |
| --- | --- | --- | --- | --- | --- | --- | --- |
| 1 | rectal swabs | - | UN | 29 | rectal swabs | - | UN |
| 2 | oral swabs | - | UN | 30 | fecal swabs | - | UN |
| 3 | rectal swabs | + | 3.23×10^2^ | 31 | oral swabs | + | 4.56×10^3^ |
| 4 | fecal swabs | - | UN | 32 | fecal swabs | - | UN |
| 5 | fecal swabs | + | 5.74×10^3^ | 33 | rectal swabs | - | UN |
| 6 | fecal swabs | - | UN | 34 | rectal swabs | - | UN |
| 7 | oral swabs | - | UN | 35 | rectal swabs | - | UN |
| 8 | oral swabs | - | UN | 36 | fecal swabs | + | 3.12×10^5^ |
| 9 | oral swabs | + | 7.85×10^1^ | 37 | oral swabs | - | UN |
| 10 | oral swabs | - | UN | 38 | rectal swabs | + | 8.26×10^1^ |
| 11 | rectal swabs | - | UN | 39 | fecal swabs | + | 2.97×10^5^ |
| 12 | oral swabs | - | UN | 40 | oral swabs | - | UN |
| 13 | oral swabs | - | UN | 41 | fecal swabs | - | UN |
| 14 | rectal swabs | - | UN | 42 | rectal swabs | + | 7.15×10^1^ |
| 15 | rectal swabs | + | 8.74×10^4^ | 43 | rectal swabs | + | 5.62×10^2^ |
| 16 | fecal swabs | - | UN | 44 | rectal swabs | - | UN |
| 17 | fecal swabs | - | UN | 45 | rectal swabs | - | UN |
| 18 | oral swabs | + | 5.48×10^6^ | 46 | fecal swabs | - | UN |
| 19 | oral swabs | - | UN | 47 | fecal swabs | - | UN |
| 20 | rectal swabs | - | UN | 48 | oral swabs | - | UN |
| 21 | fecal swabs | - | UN | 49 | fecal swabs | - | UN |
| 22 | oral swabs | - | UN | 50 | fecal swabs | - | UN |
| 23 | fecal swabs | - | UN | 51 | oral swabs | - | UN |
| 24 | rectal swabs | - | UN | 52 | rectal swabs | + | 4.83×10^6^ |
| 25 | rectal swabs | - | UN | 53 | rectal swabs | - | UN |
| 26 | fecal swabs | - | UN | 54 | oral swabs | - | UN |
| 27 | oral swabs | + | 2.15×10^2^ | 55 | rectal swabs | + | 3.82×10^2^ |
| 28 | fecal swabs | + | 7.36×10^2^ | 56 | rectal swabs | - | UN |

**Supplementary Table 2. Nucleotide sequences used in this study.**

| Sequence name | Sequence (5’—3’) |
| --- | --- |
| RAA-1F | TAATACGACTCACTATAGGGAACGAAGACATTCTAGGCAGCATAGTCATC |
| RAA-2F | TAATACGACTCACTATAGGGTTCTAGGCAGCATAGTCATCAACAACCAGA |
| RAA-3F | TAATACGACTCACTATAGGGAGACATTCTAGGCAGCATAGTCATCAACAA |
| RAA-1R | GAGGTGTAGGGGCTGACGCTACGAGCGGTT |
| RAA-2R | GGGGAGGTGTAGGGGCTGACGCTACGAGCG |
| RAA-3R | GAGTAAGAGTCAGAGTCTGGTTCTATGTCG |
| crRNA1 | CACCCGUGCAAAAUUGCAGGGGUCUAAAACACUCGGGCUCUGGCUCUGGGGACGAUGA |
| crRNA2 | CACCCGUGCAAAAUUGCAGGGGUCUAAAACUGGGGACGAUGAAGAUUAUGAAUAUGAG |
| crRNA3 | CACCCGUGCAAAAUUGCAGGGGUCUAAAACUGGCUCUGGGGACGAUGAAGAUUAUGAA |
| Probe-1 | 6-FAM-UUUUUU-BHQ |
| Probe-2 | 6-FAM-UUUUUU-Biotin |

**Supplementary Fig. 1. SDS-PAGE validation of EsCas13d expression and purification.**

Lane M: protein marker; Lane 1: negative control; Lane 2: purified EsCas13d protein.

**Supplementary Fig. 2. Visual fluorescence results of clinical samples detected by the RADIANT assay.**

**Supplementary Fig. 3. LFA detection results of clinical samples using the RADIANT assay.**
